# Critical Care Units in Malawi: A Cross-Sectional Study

**DOI:** 10.5334/aogh.4053

**Published:** 2023-08-03

**Authors:** Paul D. Sonenthal, Noel Kasomekera, Emilia Connolly, Emily B. Wroe, Martha Katete, Tadala Minyaliwa, Regan H. Marsh, Grace Banda-Katha, Mulinda Nyirenda, Kirstin W. Scott, Alice Bukhman, Joia Mukherjee, Shada A. Rouhani

**Affiliations:** 1 Brigham and Women’s Hospital, Division of Pulmonary and Critical Care Medicine, 75 Francis St, Boston, MA 02115, USA; 2 Harvard Medical School, 25 Shattuck St, Boston, MA 02115, USA; 3 Partners In Health, 800 Boylston St, Suite 300, Boston, MA 02199, USA; 4 Ministry of Health, P.O. Box 30377, Lilongwe 3, MW; 5 Abwenzi Pa Za Umoyo/Partners In Health, PO Box 56, Neno, MW; 6 University of Cincinnati College of Medicine, Division of Pediatrics, 3230 Eden Ave, Cincinnati, OH 45267, USA; 7 Cincinnati Children’s Hospital Medical Center, Division of Hospital Medicine, 3333 Burnet Ave, Cincinnati, OH 45229, USA; 8 Brigham and Women’s Hospital, Division of Global Health Equity, 75 Francis St, Boston, MA 02115, USA; 9 Brigham and Women’s Hospital, Department of Emergency Medicine, 75 Francis St, Boston, MA 02115, USA; 10 Queen Elizabeth Central Hospital, Adult Emergency and Trauma Centre, P.O. Box 95, Blantyre, MW; 11 Kamuzu University of Health Sciences, Private Bag 360, Chichiri, Blantyre 3, MW; 12 University of Washington, Department of Emergency Medicine, 325 Ninth Street, Seattle, WA, 98104, USA

**Keywords:** critical care, health systems strengthening, facility assessment, intensive care unit, high dependency unit, Malawi

## Abstract

**Background:**

The global burden of critical illness falls disproportionately outside high-income countries. Despite younger patient populations with similar or lower disease severity, critical illness outcomes are poor outside high-income countries. A lack of data limits attempts to understand and address the drivers of critical care outcomes outside high-income countries.

**Objectives:**

We aim to characterize the organization, available resources, and service capacity of public sector critical care units in Malawi and identify barriers to improving care.

**Methods:**

We conducted a secondary analysis of the Malawi Emergency and Critical Care Survey, a cross-sectional study performed from January to February 2020 at all four central hospitals and a simple random sample of nine out of 24 public sector district hospitals in Malawi, a predominantly rural, low-income country of 19.6 million in southern Africa. Data from critical care units were used to characterize resources, processes, and barriers to care.

**Findings:**

There were four HDUs and four ICUs across the 13 hospitals in the Malawi Emergency and Critical Care Survey sample. The median critical care beds per 1,000,000 catchment was 1.4 (IQR: 0.9 to 6.7). Absent equipment was the most common barrier in HDUs (46% [95% CI: 32% to 60%]). Stockouts was the most common barriers in ICUs (48% [CI: 38% to 58%]). ICUs had a median 3.0 (range: 2 to 8) functional ventilators per unit and reported an ability to perform several quality mechanical ventilation interventions.

**Conclusions:**

Although significant gaps exist, Malawian critical care units report the ability to perform several complex clinical processes. Our results highlight regional inequalities in access to care and support the use of process-oriented questions to assess critical care capacity. Future efforts should focus on basic critical care capacity outside of urban areas and quantify the impact of context-specific variables on critical care mortality.

## Introduction

Realizing the goal of Universal Health Coverage (UHC)—that all people receive the full range of quality health services without exposure to financial hardship [1]—requires a strengthening of the entire healthcare spectrum, from primary preventative services to inpatient care for critical illness. While low-income countries (LICs) have made progress to expand access to quality primary care, hospital-based services such as critical care remain limited [1].

Critical care—the recognition, monitoring, and treatment of patients with organ dysfunction or high risk of rapid deterioration and associated morbidity and mortality [2]—is a significant unmet need worldwide. The global burden of critical illness falls disproportionately outside high-income countries (HICs). For example, sub-Saharan Africa, home to approximately 14% of the world’s population, accounts for over 30% of the 11 million annual sepsis-related deaths worldwide [3]. Despite younger [4,5,6] patient populations with similar [5] or lower [6,7] disease severity, critical illness outcomes in low- and middle-income countries (LMICs) are significantly worse [5,6,7] than in HICs.

Malawi is a landlocked LIC in southern Africa with a predominantly rural population of 19.6 million. [8] District (secondary referral) and central (tertiary referral) hospitals occupy the top two tiers of Malawi’s four-tiered health system [9]. All four of Malawi’s public sector central hospitals have designated intensive care units (ICUs).

Facilities in Malawi lack many of the medications, equipment, and guidelines essential for the management of severe illnesses [10]. There is a high burden of critical illness in Malawi, with an estimated 204,568 incident cases of sepsis in 2017 [3], corresponding to 1,164 cases per 100,000 population. Critically ill patients in Malawi are relatively young, with a median patient age reported to be as low as 22 [11]. Reported rates of in-hospital mortality are 22% for hypoxemia [12] and 53% for reduced consciousness level (Glasgow Coma Score < 9) [12]. Sepsis case-fatality rates have been estimated to be as high as 75% [4], far greater than the global rate of approximately 22% [3].

A lack of data limits attempts to understand why critically ill patients outside of HICs have worse outcomes. This is particularly true for LICs, which are often left out of large international critical care studies. The few critical care studies with data from LICs are limited by an emphasis on urban academic and central referral hospitals and the use of convenience samples [11,13,14,15].

Improving critical care outcomes and progressing towards UHC requires additional data on LIC critical care resources, processes, and barriers [16]. In the near term, this data can shape the research agenda and inform national and international policymaking. Using facility-level data from the cross-sectional Malawi Emergency and Critical Care (MECC) Survey, we aimed to understand the organization, available resources, and service capacity of public sector ICUs and high-dependency units (HDUs) in Malawi and identify barriers to improving care.

## Methods

### Study design and setting

We conducted a secondary analysis of the MECC Survey, a cross-sectional study conducted from January to February 2020 assessing emergency and critical care (ECC) service readiness at all four central hospitals, in addition to a simple random sample of nine out of 24 public sector district hospitals in Malawi. The design and primary analysis of the MECC Survey are described elsewhere [17]. This secondary analysis included data from all HDUs and ICUs at the 13 facilities sampled by the MECC Survey.

Ethical approval for the MECC Survey protocol was granted by the Partners Healthcare Institutional Review Board in Boston, USA (2019P003457) and the National Health Science Research Committee in Malawi (Protocol #19/05/2346, approval number 2346).

### Instrument

The MECC Survey instrument is designed to generate estimates of critical care service readiness at the level of the hospital or hospital unit (e.g., ICU) using data collected from clinical staff informants working at each targeted hospital unit. The instrument consists of the WHO Hospital Emergency Unit Assessment Tool (HEAT) [18] with the addition of novel questions specific to critical care service delivery. Novel questions were developed using a modified Nominal Group Technique, then piloted and assessed for comprehensiveness, clarity, face validity, and reliability, the details of which are reported elsewhere [17].

The instrument uses several response structures. Signal function questions asked informants to describe the availability of a given resource or intervention on a scale of 1 to 3, with 1 indicating “generally unavailable”, 2 “some availability”, and 3 “adequate availability”. Availability was defined as how often patients in the target unit are able receive a resource or intervention within the timeframe needed for critical care.

Signal function responses of “generally unavailable” or “some availability” triggered a follow-up probe on the barriers encountered within the unit. Barriers were coded by informants and study staff into WHO HEAT categories of infrastructure, absent equipment, broken equipment, stockout, personnel, training, user fees, and opening hours. Informants were permitted to identify multiple barriers for each signal function. For frequency questions, informants were asked to respond on a scale of 1 to 5, with 1 indicating “almost never”, 2 “infrequently”, 3 “sometimes”, 4 “frequently”, and 5 “almost always”.

### Data collection

Between January 20 and February 18, 2020, the MECC Survey research team visited all 13 sampled hospitals. At each hospital, study staff administered the instrument to one administrator and 3 clinicians from each targeted unit (i.e., emergency unit, medical ward, and ICU and/or HDU, if present). To minimize the time burden, questions on staffing, protocols, cardiac monitoring, crash trolleys, social work, security, dieticians, physiotherapists, and spiritual support were only asked to one designated clinical lead at each unit. Data was collected and managed using REDCap electronic data capture tools.

Informants for the MECC Survey were clinical staff aged 18 years or older, who self-reported working for at least one month at the hospital unit of interest. All informants provided written informed consent.

The MECC defined critical care units (i.e., ICUs and HDUs) as discrete physical spaces within a hospital, dedicated to the care of critically ill patients. In Malawi, ICUs are typically in central hospitals and have the most resources and capacity; critical care units at district hospitals are generally designated as HDUs. In central hospitals, HDUs provide care for critically ill patients that are not prioritized for admission to ICUs [19]. The MECC followed individual hospital convention for classifying critical care units as ICUs or HDUs.

### Variables

Variables were analyzed and reported at the level of the hospital unit (i.e., ICU and HDU). We defined “adequate availability” at the unit level as a mean informant response >2.5 (out of 3). For yes/no questions, an item with at least two “yes” responses in a unit was considered available. For frequency questions, an item was considered present at the unit level if the mean was >4 out of 5. For barriers, participant level data was first calculated by taking the number of times each barrier was identified by the informant divided by the number of times the informant was probed for barriers (i.e., the number of times the informant responded “generally unavailable” or “somewhat available”).

Participant-level data for each barrier category (e.g., infrastructure) was calculated using the following formula:


\frac{{\sum\nolimits_{i = 1}^n {{y_i}} }}{{\sum\nolimits_{i = 1}^n {{x_i}} }}

where *n* is the total number of signal function questions; *y* represents signal function questions to which the participant responded, “generally unavailable” or “somewhat available” *and* identified the barrier category (e.g., infrastructure) when probed; and *x* represents signal function questions to which the participant responded, “generally unavailable” or “somewhat available.”

#### Level 1 ICU definition

We defined a level 1 ICU as any unit meeting nine criteria adapted from the World Federation of Societies of Intensive and Critical Care Medicine (WFSICCM): physicians with some experience in critical care available at least during the day, higher nurse to patient ratios, at least a twice daily reassessment of patients, pulse oximetry, cardiac monitoring, oxygen therapy, non-invasive support, basic quality improvement, and transfer policies [2].

#### ABCDEF bundle for mechanical ventilation

The Society of Critical Care Medicine’s ABCDEF bundle is a tool for the implementation of quality mechanical ventilation [20]. The MECC Survey included signal functions assessing elements of the bundle: assessing, preventing, and managing pain; performing spontaneous awakening trials and spontaneous breathing trials; delirium monitoring and management; early mobility and exercise; and family engagement.

### Missing data

Because resources or interventions cannot be provided in a timely manner if staff are unaware of their availability, responses of “don’t know” were coded as “generally unavailable” for signal functions. Responses of “don’t know” were coded as “missing/incomplete” for frequency questions because the frequency of an event is not dependent on clinician awareness. These same approaches were used for unit variables with one “missing/incomplete” response (i.e., data was available from only two informants). If data was missing from two or more informants in a unit, we considered unit data for the variable as “missing/incomplete”.

### Statistical analyses

Data was analyzed using Stata (Release 16). The sample size of nine district and four central hospitals was determined for the broader MECC Survey and reported separately [17]. We reported continuous and ordinal variables using medians, ranges, and interquartile ranges and categorical variables using frequencies, proportions, and 95% confidence intervals.

Despite their overlapping functions, there are substantial differences between ICUs and HDUs (e.g., ICUs are in urban tertiary centers, as opposed to HDUs, which are mainly in rural secondary hospitals). Therefore, we reported separate data for HDUs and ICUs. Comparisons of barriers between ICUs and HDUs were made with Fisher’s exact test, using a nominal level of 5% for statistical significance (two-tailed).

### Study reporting

This manuscript adheres to the reporting standards of the STROBE Statement for cross-sectional studies.

### Role of funding source

The funders had no role in study design; collection, analysis and interpretation of data; decision to publish; or preparation of the manuscript.

## Results

A total of four HDUs and four ICUs were included in MECC Survey sample of nine district hospitals and four central hospitals. All four HDUs and four ICUs were included in this analysis (Table 1). Three central hospitals had an ICU, one central hospital had an ICU and an HDU, three district hospitals had an HDU, and six district hospitals had no ICU or HDU. Unit level estimates were calculated based on the responses of three staff informants at seven (88%) units. At the one remaining unit, only two staff members were present during the MECC Survey data collection; data for the third staff informant was therefore treated as missing. Participant data was missing for an additional two (0.03%) critical care signal function responses.

**Table 1:** Characteristics of units and staff informants.


	HDUs	ICUs

**Unit characteristics**		

*n*	4	4

District hospital *n (%)*	3 (75%)	0

Central hospital *n (%)*	1 (25%)	4 (100%)

Total beds *n*	14	20

Beds per unit *median (range)*	4 (2 to 4)	4 (4 to 8)

***Patient types admitted***		

Medical adult *n (%)*	4 (100%)	4 (100%)

Medical pediatric *n (%)*	4 (100%)	4 (100%)

Surgical adult *n (%)*	3 (75%)	4 (100%)

Trauma adult *n (%)*	3 (75%)	3 (75%)

Trauma pediatric *n (%)*	3 (75%)	3 (75%)

Obstetrics *n (%)*	3 (75%)	4 (100%)

Gynecological *n (%)*	3 (75%)	4 (100%)

**Staff informant characteristics**		

*n*	11*	12

Nurse *n* (%)	6 (55%)	7 (58%)

Clinical officer *n (%)*	5 (45%)	2 (17%)

Doctor *n (%)*	0	3 (25%)

Days per week working on unit *median (IQR)*	5.0 (4.0 to 5.0)	5.0 (5.0 to 5.0)


IQR: Interquartile range. *One HDU had only two informants.

HDUs had a median 4.0 (range: 2 to 4) beds per unit, while ICUs had 4.0 (4 to 8). The median number of critical care beds (i.e., ICU and HDU beds) per 1,000,000 catchment population was 1.4 (interquartile range [IQR]: 0.9 to 6.7). Most HDUs and ICUs provided care to medical, surgical, obstetric, and trauma patients. No unit met all level 1 ICU criteria (Table S1).

All critical care units had nurses and providers present 24 hours a day (Table 2). In ICUs, there was a median 1.6 (range: 0.8 to 2.7) beds per nurse during daytime shifts compared to 2.5 (1.0 to 4.0) in HDUs. Critical care units relied primarily on a core cadre of dedicated nurses working only in the critical care unit (i.e., non-rotating). There was a median of 1.0 (0 to 2) critical care physicians available to staff ICUs and no critical care physicians in HDUs.

**Table 2:** Staff.


	HDUs (N = 4)	ICUs (N = 4)

**Units with available specialists and allied health professionals**		

Social work *n (%)*	1 (25%)	0

Radiology results interpreted by radiologist *n (%)*	1 (25%)	2 (50%)

Security *n (%)*	2 (50%)	3 (75%)

Spiritual support *n (%)*	1 (25%)	1 (25%)

Dietician *n (%)*	3 (75%)	3 (75%)

Respiratory therapist *n (%)*	1 (25%)	4 (100%)

Physical therapy *n (%)*	4 (100%)	4 (100%)

Clinical engineering *n (%)*	4 (100%)	4 (100%)

**Staff pool**		

Non-rotating nurses *median (range)*	5.0 (4 to 10)	13.5 (4 to 21)

Rotating nurses *median (range)*	1.5 (0 to 5)	1.0 (0 to 3)

Non-rotating advanced practice providers* *median (range)*	0.5 (0 to 1)	6.0 (1 to 10)

Rotating advanced practice providers *median (range)*	3.5 (0 to 6)	0 (0 to 0)

Non-rotating doctors without specialty training *median (range)*	0 (0 to 1)	0 (0 to 3)

Rotating doctors without specialty training *median (range)*	2.0 (0 to 3)	0 (0 to 3)

Non-rotating physicians specializing in critical care *median (range)*	0 (0 to 0)	1.0 (0 to 2)

Rotating physicians specializing in critical care *median (range)*	0 (0 to 0)	0 (0 to 0)

Non-rotating physicians with other specialization *median (range)*	0 (0 to 0)	0 (0 to 0)

Rotating physicians with other specialization *median (range)*	0 (0 to 3)	0 (0 to 3)

**Coverage**		

Units with nurses available 24 hours/day *n (%)*	4 (100%)	4 (100%)

Units with providers physically present in unit 24 hours/day *n (%)*	4 (100%)	4 (100%)

Number of beds per nurse during the daytime *median (range)*	2.5 (1.0 to 4.0)	1.6 (0.8 to 2.7)

Number of beds per nurse overnight *median (range)*	3.0 (2. 0 to 4.0)	2.3 (1.0 to 4.0)

Agreement with reported staffing numbers for nurses and number of nurses working at time of visit *n (%)*	3 (75%)	1 (25%)


Non-rotating: dedicated staff working only in the critical care unit. ^*^ Advanced practice providers include clinical officers and anesthetists.

All HDUs and two (50%) of the ICUs had written policies outlining criteria for admission to the unit (Table S3). Staff reported checking vital signs at regular intervals every 1.3 (1 to 2) hours at HDUs and 2.0 (2 to 2) hours at ICUs. The availability of condition-specific treatment protocols was variable. Half of all critical care units had protocols for asthma and pneumonia treatment. Although a sepsis protocol was available at three (75%) HDUs and three (75%) ICUs, a volume resuscitation protocol was available at one (25%) HDU and three (75%) ICUs. Protocols for end-of-life care were available at two (50%) HDUs and no ICUs.

Aside from peripheral intravenous cannulas, which all critical care units could place, options for venous access were limited. Only one (25%) HDU and two (50%) ICUs could establish central venous access, and no units were able to place an intraosseous device or perform venous cutdown. (Of note, ICUs and HDUs reported the presence of national policies restricting both procedures). One (25%) HDU and three (75%) ICUs were able to administer intravenous vasopressors (including adrenaline).

Except for one HDU, all critical care units could perform bag-valve-mask ventilation (Table 3). All ICUs but no HDUs were capable of non-invasive and invasive ventilation. However, two (50%) HDUs had the capability of intubating patients and transferring them to a higher level of care for mechanical ventilation. The median number of functional ventilators per ICU was 3.0 (range: 2 to 8), with a median 0.9 (0.4 to 1.3) ventilators per 1,000,000 ICU catchment population (Table 4). No ICUs had written policies for the initiation or withdrawal of mechanical ventilation. All ICUs were able to maintain the head of bed elevated to prevent ventilator-associated pneumonia, and three (75%) ICUs could administer and maintain neuromuscular blockade. Only one (25%) ICU reported the availability of blood gas analysis.

**Table 3:** Critical care signal functions.


	HDUs (N = 4)	ICUs (N = 4)

**Airway and breathing**		

Bag-valve-mask ventilation *n (%)*	3 (75%)	4 (100%)

Surgical airway *n (%)*	1 (25%)	3 (75%)

Placement of supraglottic airway *n (%)*	0	2 (50%)

Endotracheal intubation *n (%)*	2 (50%)	4 (100%)

Non-invasive ventilation *n (%)*	0	4 (100%)

Invasive mechanical ventilation *n (%)*	0	4 (100%)

**Cardiac and circulation**		

Administer aspirin for ischemia *n (%)*	4 (100%)	2 (50%)

Perform external defibrillation and/or cardioversion *n (%)*	0	4 (100%)

Administer adrenaline *n (%)*	3 (75%)	3 (75%)

Administer intravenous vasopressors *n (%)*	1 (25%)	3 (75%)

Administer inotropes *n (%)*	2 (50%)	3 (75%)

Administer anti-arrhythmics *n (%)*	0	1 (25%)

Administer thrombolytics *n (%)*	0	1 (25%)

**Procedures**		

Place peripheral intravenous cannula *n (%)*	4 (100%)	4 (100%)

Establish central venous access *n (%)*	1 (25%)	2 (50%)

Establish intraosseous access *n (%)*	0	0

Perform venous cutdown *n (%)*	0	0

Perform paracentesis *n (%)*	4 (100%)	2 (50%)

Perform lumbar puncture *n (%)*	4 (100%)	4 (100%)

Placement of chest tube *n (%)*	3 (75%)	3 (75%)

Needle decompression of pneumothorax *n (%)*	2 (50%)	2 (50%)

Perform pericardiocentesis *n (%)*	0	0

**Supportive care**		

Administer enteral nutrition *n (%)*	4 (100%)	4 (100%)

Frequently (at least every four hours) check electrolytes and adjust management based on results *n (%)*	1 (25%)	1 (25%)

Regularly (at least every four hours) reposition patients to prevent pressure ulcers *n (%)*	4 (100%)	4 (100%)

Administer stress ulcer prophylaxis *n (%)*	0	3 (75%)

Administer DVT prophylaxis *n (%)*	2 (50%)	3 (75%)

Provide physical restraints *n (%)*	4 (100%)	4 (100%)

Manage extreme temperatures *n (%)*	4 (100%)	4 (100%)

De-escalate care (e.g., stop treatments or remove life support) for patients with poor prognoses based on the expressed goals and wishes of the patient or their families *n (%)*	0	1 (25%)


DVT: deep vein thrombosis.

**Table 4:** Mechanical ventilation in ICUs.


	ICUs (N = 4)

Functional ventilators per unit *median (range)*	3.0 (2 to 8)

Vents per 1,000,000 population in catchment area *median (range)*	0.9 (0.4 to 1.3)

Maintain head of bed in semi-recumbent position (30-45 degrees) to reduce aspiration and ventilator associated pneumonia *n (%)*	4 (100%)

Administer and maintain neuromuscular blockade *n (%)*	3 (75%)

Written policy for who can or cannot be intubated and/or placed on mechanical ventilation *n (%)*	0

Written policy for withdrawal of mechanical ventilation *n (%)*	0

ABCDEF bundle*	

*Assess, prevent, and manage pain*	

Assess pain in mechanically ventilated patients at least twice a day *n (%)*	3 (75%)

Administer intravenous opioids *n (%)*	4 (100%)

*Spontaneous awakening trials and spontaneous breathing trials*	

Perform daily spontaneous breathing trials *n (%)*	3 (75%)

Perform daily spontaneous awakening trials *n (%)*	3 (75%)

*Analgesia and sedation*	

Assess agitation/sedation in mechanically ventilated patients at least twice a day *n (%)*	3 (75%)

Administer appropriate therapeutics for agitation *n (%)*	4 (100%)

Administer intravenous sedatives *n (%)*	4 (100%)

*Delirium: Assess, prevent, and manage*	

Assess delirium in mechanically ventilated patients at least twice a day *n (%)*	1 (25%)

*Early mobility and exercise*	

Perform early mobilization for mechanically ventilated patients *n (%)*	4 (100%)

*Family engagement and empowerment*	

Communicate with patient and/or families, including sharing poor prognoses *n (%)*	4 (100%)


*The Society of Critical Care Medicine’s ABCDEF bundle includes: assess, prevent, and manage pain; perform spontaneous awakening trials and spontaneous breathing trials; delirium monitoring and management; early mobility and exercise; and family engagement [26].

ICUs could perform most signal functions from the ABCDEF mechanical ventilation bundle (Table 4). All ICUs could administer intravenous opioids, therapeutics for agitation, and intravenous sedatives. Three (75%) ICUs were able to assess pain, perform spontaneous awakening and breathing trials, and assess agitation and sedation. Only one (25%) ICU was able to regularly assess for delirium.

**Figure 1:**
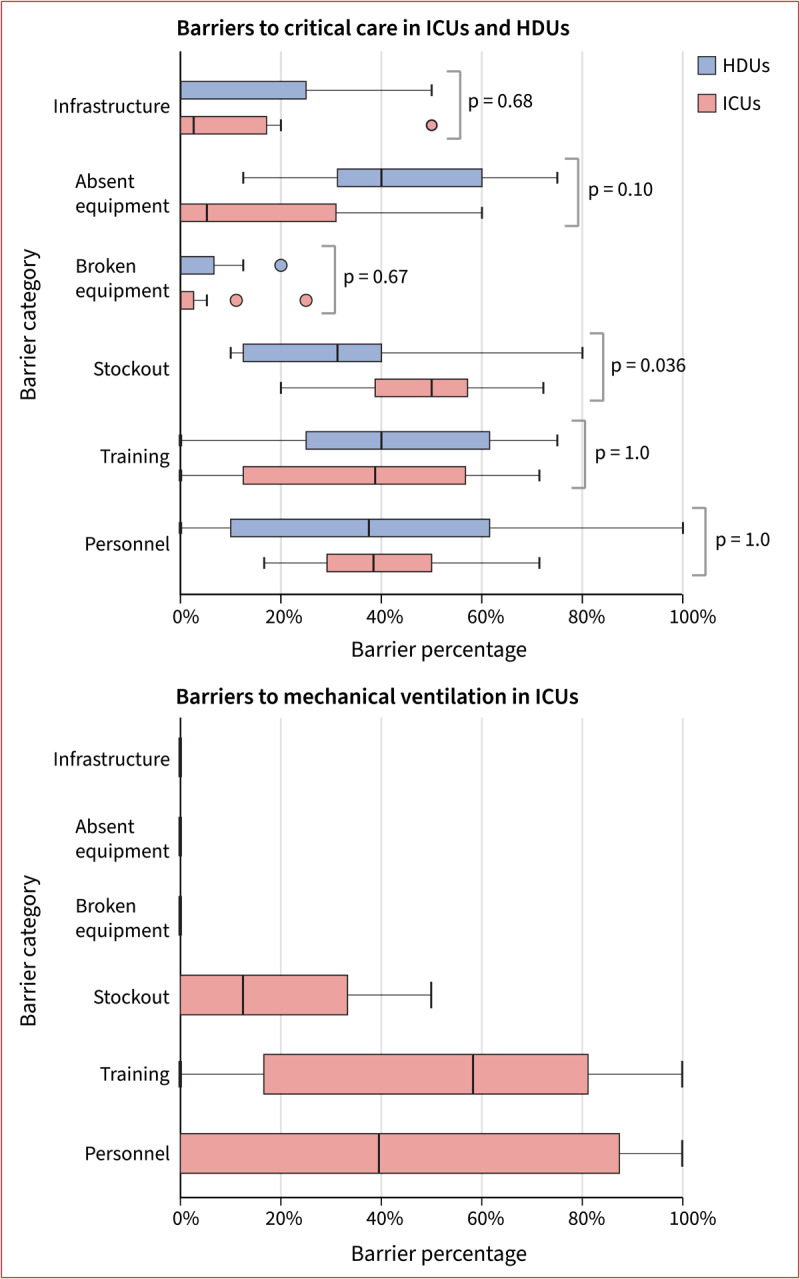
**Barriers in critical care units:** Box plots of participant level data showing relative frequencies of the six most common barrier categories in HDUs (n = 11). and ICUs (n = 12) for signal functions from Table 3 (top panel) and Table 4 (bottom panel). Estimates for each participant were calculated taking the number of times each barrier was identified by the participant divided by the number of times the participant was probed for barriers (i.e., number of times participant responded generally unavailable or somewhat available). Reported p-values are for Fisher’s exact test.

For critical care signal functions in Table 3, absent equipment was the most common barrier in HDUs (46% [95% confidence interval (CI): 32% to 60%]) but not in ICUs (16% [CI: 3% to 29%], p-value 0.10) (Figure 1). ICUs reported stockouts as a barrier (48% [CI: 38% to 58%]) more often than HDUs (33% [CI: 19% to 47%], p-value 0.036). For mechanical ventilation signal functions in Table 4, frequent barriers were personnel (52% [CI: 20% to 83%]) and training (44% [CI: 7% to 82%]). ICUs also identified stockouts (18% [CI: 1% to 35%]) as a barrier—primarily medications.

## Discussion

This study of Malawi’s public sector ICUs and HDUs identified several strengths, including nurse staffing and the ability to perform a variety of core critical care processes. We also found a need for an increased availability of critical care specialists, clinical protocols and policies, and adequate equipment and supplies, particularly in HDUs. This information has the potential to shape policymaking, care delivery, and the critical care research agenda outside of HICs.

Critical care unit capacity in Malawi is limited with only 20 ICU and 14 HDU beds across the MECC Survey sample of all four full-service public sector central hospitals and nine of 24 district hospitals. Despite reported capabilities—such as cardiac monitoring, administration of vasopressors, regular clinician reassessment, and routine patient repositioning—no unit met all the criteria for a level 1 ICU. Although the WFSICCM ICU criteria is designed to be adjusted based on context-dependent factors [2], several important elements (e.g., policies for transfer, quality improvement programs) were absent in many units.

Resources and capacity were disproportionately lower in HDUs; several lacked essentials, such as electricity, running water, and oxygen. While it may be unsurprising that ICUs have greater capabilities than HDUs, the degree of disparity raises concerns about access to care. The urban distribution of ICUs is misaligned, with 82% of Malawians residing rural areas [8]—meaning the most equipped critical care units are distant from the majority of the population. Although concentrating certain complex and resource-intensive critical care services at central hospitals makes sense, district hospitals in Malawi lack many of the basic services essential for the critical care that should be available at all hospital levels [21].

In terms of human resources, critical care units in Malawi were relatively well-staffed with nurses. The daytime bed-to-nurse ratio of 1.6 in Malawian ICUs is in line with other national and international assessments [22,23,24], although they lack physician critical care specialists. This pattern is consistent with other published comparisons of LMIC and HIC ICUs [15,25,26]. The ongoing development of the nursing workforce and the creation of opportunities for physician critical care specialization are needed.

The presence of condition-specific protocols, including for common conditions, such as asthma, pneumonia, and diabetic ketoacidosis was inconsistent. ICU-specific protocols were also variably present, with only 50% of ICUs with formal admission criteria. Similar findings have also been reported in Tanzania (50%) [13] and Uganda (17%) [23].

Although barriers were identified across all categories, absent equipment and stockouts were the most common barriers identified in HDUs and ICUs, respectively. This diverges from prior LIC critical care data, which identified equipment and commodity availability as relative strengths [13,14]. While these differences could be context-specific, methodology may also play a role. Signal functions interrogated the availability of processes, rather than the physical presence of an item. The presence of a functional glucometer does not guarantee the ability to check glucose if the glucometer is shared across several units, or if replacement batteries are difficult to obtain. This distinction is of particular importance in critical care, where rapid access to interventions is essential.

Critical care often includes caring for dying patients. This includes ensuring patient comfort and dignity and discontinuing aggressive interventions (e.g., mechanical ventilation) when inconsistent with the expressed goals of the patient or family. None of the ICUs included in the MECC Survey had a written policy guiding the withdrawal of mechanical ventilation, and only one reported the ability to de-escalate care in these situations. This is striking, considering that in one Malawian ICU, nearly 10% of all ICU admissions are diagnosed with brain death, yet all receive intensive care until cardiac death [27].

Mechanical ventilation is one of the most complex interventions provided in critical care units, requiring detailed quality processes reliant on the availability of equipment, reliable infrastructure, meticulous expert nursing care, and well-trained clinicians. ICUs in this study had 0.9 ventilators per 1,000,000 catchment population. By comparison, Ghana has 1.7 [22], The Gambia 1.5 [25], and the United States 197 [28].

Despite a limited numbers of ventilators, Malawian ICUs reported the ability to perform several processes central to high quality mechanical ventilation, such as spontaneous breathing and awakening trials. However, one ICU study from Malawi estimated a 29.8% mortality for mechanically ventilated patients [11]. Similarly, a pooled analysis of four large observational studies found 50% higher mechanical ventilation mortality in middle-income countries compared to HICs, despite similar rates of low volume ventilation [5]. Translating quality clinical processes into good patient outcomes requires characterizing context-specific barriers [29]. In our study, personnel and training were the most common barriers related to mechanical ventilation. This finding may only be partially explained by a lack critical care specialists. Nurse training, which would not be reflected in nursing ratios, is also likely a factor. Additionally, only one ICU in Malawi’s public sector had adequate access to blood gas analysis—an important tool for the management of mechanical ventilation. The limited availability of blood gas analysis may help explain why—despite the reported presence of several quality clinical processes—mechanical ventilation outcomes remain poor.

### Significance/future directions

This study provides additional evidence of the global disparity in access to critical care and highlights the need for a comprehensive approach to strengthening basic critical care services across all levels of the health system. For LICs with predominantly rural populations, this must include an emphasis on equipping district hospitals so they can rapidly identify and stabilize critically ill patients and provide access to safe transfer services to higher level facilities, as appropriate. Opportunities for postgraduate critical care physician training and ongoing nursing training are a priority. Yet, even with specialized training, critical care staff are unable to effectively treat patients without the necessary equipment and resources. HDUs may need upfront investment and procurement, and ICUs would benefit from an ongoing strengthening of the supply chain and logistics.

A research agenda should focus on identifying and measuring factors that determine critical care outcomes. Commentators have advocated for a multifaceted approach (clinical processes, unit structure and staffing, resources, barriers, disease type and severity, sociodemographic information, and other context-specific variables) [29,30], which our data supports. This can be accomplished through the repeated administration of the MECC Survey instrument, potentially supplemented with process observation and patient-level data, including outcomes. This will also facilitate tailored interventions and the measurement of impact. Finally, assessments of the ethical, cultural, legal, and clinical aspects of end-of-life care are necessary to develop context-specific frameworks for de-escalating care for patients who are unlikely to benefit, when appropriate.

### Limitations

This study has several limitations. Data from ICUs in Malawi may not be generalizable to other LICs. The facility sample, calculated for a broader study, included all public central hospital ICUs, but was relatively small. This study aimed to characterize publicly accessible critical care in Malawi and therefore, only sampled government facilities. Although scarce, there are at least two non-governmental ICUs in Malawi [31], and their exclusion may have impacted our results. Administration of the survey in person may have introduced reporting bias, leading to the overestimation of resources and the ability to perform complex processes, including elements in the ABCDEF mechanical ventilation bundle. It is also important to recognize that not all critical care takes place in ICUs and HDUs, although it is reasonable to assume that critical care units provide the highest level of critical care available in each facility.

## Conclusion

These findings provide a detailed description of critical care, including the resources, processes, and barriers in public sector HDUs and ICUs in Malawi. Although many gaps are present, HDUs and ICUs reported the ability to perform multiple complex critical care processes. Our results highlight both international and regional disparities and raise the possibility that process-oriented questions could provide more accurate information about critical care service availability. Future efforts should be directed at strengthening essential critical care capacity outside of urban areas and quantifying the impact of context-specific variables on mortality from critical illness.

## Additional File

The additional file for this article can be found as follows:

10.5334/aogh.4053.s1 Supplemental Tables. Tables S1 to S3.
