# Supplementary material for: Critical Care Units in Malawi: A Cross-Sectional Study
Source: Ann Glob Health. 2023 Aug 3;89(1):51. doi: 10.5334/aogh.4053 (PMC10402812; doi:10.5334/aogh.4053)
Supplement: Supplemental Tables. — Tables S1 to S3. [file agh-89-1-4053-s1.pdf]

Supplement to: Critical Care Units in Malawi: A Cross-Sectional Study

Paul D Sonenthal, MD[1,2,3]; Noel Kasomekera, MPH[4,5]; Emilia Connolly, DO, MPH[5,6,7]; Emily B Wroe, MD, MPH[2,5,8]; Martha Katete, BSN[5]; Tadala Minyaliwa, MBBS[5]; Regan H Marsh, MD, MPH[2,3,9]; Grace Banda-Katha, MBBS, FCEM[10]; Mulinda Nyirenda, MBBS, MMed, FCP[10,11]; Kirstin W Scott, MD, PhD[12]; Alice Bukhman, MD, MPH[2,8]; Joia Mukherjee, MD, MPH[2,3]; Shada A Rouhani, MD, MPH[2,3,8]

[1]Brigham and Women's Hospital, Division of Pulmonary and Critical Care Medicine, 75 Francis St, Boston, MA 02115, USA
[2]Harvard Medical School, 25 Shattuck St, Boston, MA 02115, USA
[3]Partners In Health, 800 Boylston St, Suite 300, Boston, MA 02199, USA
[4]Ministry of Health, P.O. Box 30377, Lilongwe 3, Malawi
[5]Abwenzi Pa Za Umoyo/Partners In Health, PO Box 56, Neno, Malawi
[6]University of Cincinnati College of Medicine, Division of Pediatrics, 3230 Eden Ave, Cincinnati, OH 45267, USA
[7]Cincinnati Children's Hospital Medical Center, Division of Hospital Medicine, 3333 Burnet Ave, Cincinnati, OH 45229, USA
[8]Brigham and Women's Hospital, Division of Global Health Equity, 75 Francis St, Boston, MA 02115, USA
[9]Brigham and Women's Hospital, Department of Emergency Medicine, 75 Francis St, Boston, MA 02115, USA
[10]Queen Elizabeth Central Hospital, Adult Emergency and Trauma Centre, P.O. Box 95, Blantyre, Malawi
[11]University of Malawi College of Medicine, Private Bag 360, Chichiri, Blantyre 3, Malawi
[12]University of Michigan, 1500 E. Medical Center Drive, Ann Arbor, Michigan, 48109, USA

TABLE S1. LEVEL 1 ICU CRITERIA 2

TABLE S2. INFRASTRUCTURE, EQUIPMENT, AND DIAGNOSTICS 3

TABLE S3. PRACTICE PATTERNS AND CLINICAL PROTOCOLS 4

Corresponding author contact information
Paul D. Sonenthal, MD
Brigham and Women's Hospital, Division of Pulmonary and Critical Care Medicine
Email: psonenthal@pih.org

Table S1. Level 1 ICU criteria

	HDUs (n=4)	ICUs (n=4)
Physicians with some experience in critical care available, at least during the day *n (%)*	0	3 (75%)
Higher nurse to patient ratios for critically ill patients *n (%)*	4 (100%)	4 (100%))
At least twice daily reassessment of critically ill patients *n (%)*	2 (50%)	2 (50%)
Pulse oximetry *n (%)*	2 (50%)	4 (100%)
Cardiac monitoring *n (%)*	3 (75%)	4 (100%)
Availability of oxygen *n (%)*	2 (50%)	4 (100%)
Availability of non-invasive ventilation *n (%)*	0	4 (100%)
Basic quality improvement program^ *n (%)*	4 (100%)	2 (50%)
Policy and protocol for transferring patients to higher level of care *n (%)*	1 (25%)	0
Number of criteria met (out of a possible 9) *median (range)*	4.5 (3 to 6)	6.0 (5 to 7)
Units meeting all Level 1 ICU criteria *n (%)*	0	0

Criteria adapted from the World Federation of Societies of Intensive and Critical Care Medicine
^Defined as any quality improvement project related to the ward within the last 12 months.

Table S2. Infrastructure, equipment, and diagnostics

	HDUs (n=4)	ICUs (n=4)
Infrastructure		
Running water *n (%)*	3 (75%)	4 (100%)
Electricity *n (%)*	2 (50%)	4 (100%)
Staff work area *n (%)*	0	0
Toilet for patient and staff *n (%)*	2 (50%)	4 (100%)
Storage space (including substances) *n (%)*	3 (75%)	4 (100%)
Crash trolley or code cart *n (%)*	2 (50%)	4 (100%)
Oxygen sources		
Central piped system *n (%)*	1 (25%)	1 (25%)
Concentrator stored on unit *n (%)*	2 (50%)	3 (75%)
Tanks stored on unit *n (%)*	2 (50%)	4 (100%)
Call for tank from central location if needed *n (%)*	2 (50%)	4 (100%)
Call for concentrator from central location if needed *n (%)*	2 (50%)	4 (100%)
None n (%)	2 (50%)	0
Diagnostics and monitoring		
Full blood count *n (%)*	4 (100%)	4 (100%)
Glucose *n (%)*	3 (75%)	4 (100%)
Electrolytes *n (%)*	2 (50%)	2 (50%)
Blood urea nitrogen and creatinine *n (%)*	2 (50%)	4 (100%)
Lipase *n (%)*	0	0
Coagulation profile (PT/PTT, INR) *n (%)*	0	0
Cross matching for blood and blood products *n (%)*	4 (100%)	4 (100%)
Cardiac marker (e.g., troponin) *n (%)*	0	0
Arterial blood gas *n (%)*	0	1 (25%)
Perform and interpret electrocardiogram *n (%)*	2 (50%)	4 (100%)
Perform and interpret point of care ultrasound *n (%)*	2 (50%)	3 (75%)
Stationary x-ray *n (%)*	4 (100%)	3 (75%)
Portable x-ray *n (%)*	1 (25%)	2 (50%)
CT scan *n (%)*	0	1 (25%)

Table S3. Practice patterns and clinical protocols

	HDUs (n=4)	ICUs (n=4)
Formal written policy outlining criteria for admission to unit *n (%)*	4 (100%)	2 (50%)
Formal written policy outlining criteria for discharge from unit *n (%)*	2 (50%)	3 (75%)
Frequency (in hours) of observations for patients *median (range)*	1.2 (1 to 2)	2.0 (1 to 2)
Frequency (in hours) of vital signs *median (range)*	1.3 (1 to 2)	2.0 (2 to 2)
Nurse can initiate treatment in response to clinical change *n* (%)	4 (100%)	4 (100%)
Protocols		
Volume resuscitation *n (%)*	1 (25%)	3 (75%)
Asthma treatment *n (%)*	2 (50%)	2 (50%)
Pneumonia treatment *n (%)*	2 (50%)	2 (50%)
Sepsis treatment *n (%)*	3 (75%)	3 (75%)
Diabetic ketoacidosis *n (%)*	3 (75%)	2 (50%)
Infection control *n (%)*	3 (75%)	3 (75%)
Managing hazardous exposures (including designated decontamination area) *n (%)*	4 (100%)	2 (50%)
Containment and disposal of sharps and biomedical waste *n (%)*	4 (100%)	3 (75%)
Protocol for post exposure prophylaxis for healthcare workers *n (%)*	3 (75%)	3 (75%)
Hand-over protocols when transferring patients from one care provider to another *n (%)*	2 (50%)	3 (75%)
End-of-life care *n (%)*	2 (50%)	0
Monitoring of nosocomial infections and antimicrobial resistance patterns *n (%)*	0	0
